# Life Course Pathways Into Intergenerational Caregiving

**DOI:** 10.1093/geronb/gbac024

**Published:** 2022-02-05

**Authors:** Ricardo Rodrigues, Maša Filipovič Hrast, Selma Kadi, Miriam Hurtado Monarres, Valentina Hlebec

**Affiliations:** European Centre for Social Welfare Policy and Research, Vienna, Austria; University of Ljubljana Faculty of Social Sciences, Ljubljana, Slovenia; European Centre for Social Welfare Policy and Research, Vienna, Austria; University of Ljubljana Faculty of Social Sciences, Ljubljana, Slovenia; University of Ljubljana Faculty of Social Sciences, Ljubljana, Slovenia

**Keywords:** Austria, Cumulative advantage/disadvantage, Dyads, Qualitative methods, Slovenia

## Abstract

**Objectives:**

We develop a framework for the analysis of pathways into intergenerational caregiving to older people provided by family members using life course concepts of key turning events in life, cumulative processes, and linked lives within the family realm.

**Methods:**

Using framework analysis, we analyze semistructured qualitative interviews from a sample of dyads (older cared-for adults and their main family carers comprised of children, children-in-law, and grandchildren) in Austria (*N* = 24) and Slovenia (*N* = 52). Data were collected in 2019 through purposive sampling, including dyads from a differentiated socioeconomic background and gender.

**Results:**

The analysis reveals 4 nonexclusive pathways into caregiving. One pathway is associated with single turning events occurring in family or work trajectories of carers that expanded the possibilities for caregiving later in life. A second pathway referred to cumulative processes that later influenced transitions into caregiving, such as personal biographies marked by weak labor market attachment. Another cumulative pathway, exclusive to caregiving, is characterized by continued and sustained exchanges of support within families that cement reciprocal ties that underpin caregiving at later stages. In the fourth pathway, life trajectories of siblings, but also family relationships and conflicts, constrained carers into their role.

**Discussion:**

Decisions regarding caregiving within families can be best understood as processes, linked to developments in other trajectories in carers’ lives, as much as to internal family dynamics and relationships. Becoming a carer may be itself the result of intertwined accumulated vulnerabilities, as well as cumulative exchanges within families.

Intergenerational caregiving provided by family members makes up for a substantial share of care provided to older people in Europe and North America and its relevance seems unlikely to diminish in the future (Barczyk & Kredler, 2019). While the determinants of intergenerational family caregiving—that is, care provided by children, children-in-law, and grandchildren—have been widely studied across different long-term care systems (Roth et al., 2009; Schmid et al., 2012), existing research has often taken a static view of these determinants and of the timing of care transitions. Two recent reviews have questioned whether these snapshots taken at particular moments of life fully and adequately capture the complexity of caring trajectories, particularly the determinants of caregiving, and have explicitly called for a life course perspective on caregiving (Larkin et al., 2019; Moen & DePasquale, 2017).

Intergenerational family caregiving has recently merited attention as a specific trajectory of the life course. Most studies have focused on either the timing and sequence of caregiving careers (Fast et al., 2020; Keating et al., 2019) or on the impact of such caregiving careers on other life trajectories. Examples of the latter are studies analyzing how the timing of caregiving shapes further possibilities for education and employment among carers (Hamilton & Adamson, 2013; King & Pickard, 2013; Michaud et al., 2010). Transitioning into caregiving has also been shown to affect perceived family relations, that is, family life trajectories, among caregiving partners in old age (Kramer & Lambert, 1999).

In comparison, linking earlier life trajectories with transitions into caregiving later in life, for example, through accumulation or pathway processes (Graham, 2002), remains a relatively unexplored aspect. To our knowledge, only Carmichael and Ercolani (2016) specifically address informal caregiving as part of a cumulative process associated with prior life events. Using longitudinal data, they show that differences in economic resources, health, and well-being well before the first episode of caregiving are associated with different patterns of informal caregiving observed later in life. Hamilton and Adamson (2013) also discuss pathways into care, more concretely the “being born into care” pathway, albeit in the context of young family carers. Our study makes a contribution toward establishing intergenerational family care, or henceforth simply family care, as a trajectory of its own in the life course by focusing on transitions into family care. This research has the potential to uncover prior or ongoing cumulative processes of inequality or disadvantage observed in carers at the time of care provision, including what is usually coined as “self-selection into caregiving” (Van Houtven et al., 2013). To this end, we propose a framework for analyzing transitions into family caregiving for older relatives which draws from life course theories and allows it to be interwoven with other life trajectories, such as family relations or labor market. We then go on to apply this framework to the analysis of a qualitative data set gathered from dyads of older adults receiving a mix of care services and informal care and their family carers in Austria and Slovenia and to adapt the framework based on the data analyzed.

## A Life Course Framework for Transitions Into Intergenerational Family Caregiving

The framework detailed below builds on four main components or concepts taken from life course: pathways, key turning events, cumulative processes, and linked lives. The framework links family caregiving later in life with events and processes starting or taking place in earlier life stages, as well as shared relationships with others (e.g., family members). In doing so, it moves away from snapshots of individual-level predictors of caregiving, to offer an alternative conceptualization based on pathways into family caregiving that are represented as dynamic life course processes within broader family constellations. The framework focuses not only on the processes but also on the contexts (e.g., family relationships, cultural norms, public policies) that underpin caregiving.

### Life Events and Transitions

The life course has been characterized as a sequence of episodes in which earlier life events influence later ones (Alwin, 2012). These events have the potential to mark bookends of life stages and transitions in the trajectories of individuals (Elder, 1985). Transitions may be discrete and relatively abrupt or gradual. In addition, the consequences of transitions in one domain of the life course are seldom circumscribed to that domain only. Life course also emphasizes the social and institutional embeddedness of trajectories, as the meaning and consequences of particular transitions are moderated by gender and socioeconomic position, for example (Moen & Sweet, 2004). Finally, the timing of life events is also key, as shown by the impact that unemployment in early youth has on lifelong earnings (Genda et al., 2010).

We posit that transitions into family caregiving are informed by other life events in different domains of the life course (e.g., family life or employment) and their timing. Transitions caused by said events, such as divorces leading to cohabitation with parents (family life), have the potential to shape life trajectories and put them in a pathway that leads to caregiving at a later stage in life.

### Cumulative Processes

Another key tenet of life course analysis is the concept of cumulative advantage/disadvantage (CAD) that attributes current individual heterogeneity within cohorts to cumulative processes (see Crystal & Shea, 1990; Dannefer, 2003; O’Rand, 1996, or more recently Crystal et al., 2017; Dannefer, 2020 on cumulative processes in aging). While these may be triggered by specific life events, CAD theory emphasizes the interaction of institutional arrangements (i.e., how opportunities are structurally constrained) and individual behavior (i.e., individual agency) in reinforcing advantages/disadvantages, in what is known as “The Mathew Effect” (Merton, 1968). O’Rand (1996) refers to women’s trajectories in the family and employment domains in the context of systems that emphasize the work-nexus for accumulation of resources as an example of the operation of such cumulative disadvantage processes. While many such cumulative processes have been defined around early life or childhood experiences, they may also take place during adulthood along the lines of what Dannefer (2020) coined as “life course reflexivity.” Transitions into caregiving may thus result from a cumulative process of disadvantage during adulthood, for example, in weak labor market attachment or limited accumulation of financial resources that leads to caregiving.

### Linked Lives

Life course scholars have long emphasized how life trajectories and individual welfare are linked to the choices, resources, and transitions of other individuals in an interdependent fashion (Alwin, 2012; Elder et al., 2003; Keating et al., 2019). In fact, this interdependency arises from the sharing of relationships between people (Alwin, 2012). A relevant concept connected to linked lives in caregiving is that of “social convoys” proposed by Kahn and Antonucci (1980) as networks of relatives and friends that can be activated to become “care convoys” when people are unable to live without assistance, that is, when needs arise (Keating et al., 2019). In our framework, we propose to look at linked lives in the sense of how transitions into caregiving are shaped by evolving relationships within the family realm. Incorporating linked lives into the framework also highlights how individuals’ agency is constrained by diverse social structures (Alwin, 2012).

## Data and Methods

### Study Design

This was a qualitative study carried out in Austria and Slovenia and based on semistructured face-to-face interviews with dyads of older adults living in the community and their self-identified primary family carer (i.e., children, children-in-law, or grandchildren of the person cared-for). For the purpose of the analysis, we defined transition into caregiving in relation to a single still ongoing care episode (i.e., with a beginning, but not yet completed at the time of the interview). The choice of qualitative research methods allows us to better explore the interviewees’ own perceptions of the transitions into caregiving and gain an in-depth knowledge of their biographies and key transitions in life. Drawing on Eiskovits and Koren (2010), we selected to interview dyads to better capture the relational aspects involved in caregiving, namely, the evolving relationships within families, but also how events and transitions taking place in the life trajectories of one dyad member affected the other one or the dyad as a whole (i.e., their relationship). The dyadic nature of the study also allowed us to systematically contrast similarities and differences in the accounts or significance of transitions and evolving family relationships.

The countries selected represent a most-similar study design (Landman, 2008) as they are neighboring countries with similar predominant care values and approaches to care provision that in both cases are closer to the familialism side of the care regime spectrum (Saraceno, 2016). Austria and Slovenia share strong family values that emphasize the family as the main caregiver (Eurobarometer, 2007). In both countries, carers live relatively close to their dependent relatives as housing mobility is low, with a similar share (25%–30%) of older adults living alone (Eurostat, 2015). At the same time, dyads were selected to have a diverse socioeconomic status and gender—in effect a most-dissimilar study design concerning interviewees (Landman, 2008)—to capture a wide variation of pathways into informal caregiving.

### Sampling and Recruiting

The study included 52 dyads in Slovenia and 24 in Austria. Potential interviewees were selected among people aged 60 and older who were receiving care services and informal care and whose cognitive capacity allowed them to be interviewed. This meant that they had been assessed as in need of care and support in Slovenia and were receiving at least Level 2 of the cash-for-care benefit in Austria (i.e., assessed as needing at least 95 h of care per month). Potential interviewees were contacted through home care service providers in each country, community services, and organizations (e.g., General Practitioner (GP) and religious organizations), as well as informal contacts as part of snowball sampling. The interviewees were purposely sampled to ensure a diverse representation of socioeconomic status (SES) and gender of both care recipients and caregivers (Table 1). The SES of care recipients was defined around education and income, while for informal carers it was defined around occupation and education (see the footnote in Table 1). The level of care needs was proxied by the frequency of care services received, as well as by information on the type of care received (available upon request from the authors) and was relatively high on the whole sample. To minimize recall bias, shortlisted interviewees were limited to those who had been using care services for no longer than 12 months, a threshold that was later extended to 24 months. Approval for the study was obtained from the Ethics Committee at the Faculty of Social Sciences, University of Ljubljana (2016-01/KERFDV) as no ethical approval is required for this type of study in Austria, and all participants signed a written consent form to participate.

**Table 1.** Characteristics of the Interviewed Dyads in Each Country

	Caregiver			Cared-for person		
	Austria	Slovenia	Total (%)	Austria	Slovenia	Total (%)
Gender						
Male	7	22	29 (38.2)	5	8	13 (17.1)
Female	17	30	47 (61.8)	19	44	63 (82.9)
Socioeconomic status						
Low	5	5	10 (13.2)	8	29	37 (48.7)
Middle	10	21	31 (40.8)	11	16	27 (35.5)
High	9	26	35 (46.0)	5	7	12 (15.8)
Age group						
<50	5	20	25 (32.9)	—	—	—
50–59	10	18	28 (36.8)	—	—	—
60–74	8	14	22 (28.9)	1	7	8 (10.5)
75–84	1	—	1 (1.3)	7	18	25 (32.9)
>84	—	—	—	16	27	43 (56.6)
Employment status (caregivers)						
Full-time	14	38	52 (68.4)	—	—	—
Part-time	4	2	6 (7.9)	—	—	—
Unemployed/sick leave	—	2	2 (2.6)	—	—	—
Retired	6	10	16 (21.1)	—	—	—
Living arrangement						
Alone	10	2	12 (15.8)	19	15	34 (44.7)
With spouse or other relatives	14	50	64 (84.2)	5	37	42 (55.3)
Frequency of care services received (users)						
Daily	—	—	—	17	40	47 (75.0)
More than once a week	—	—	—	4	9	13 (17.1)
Weekly or less	—	—	—	3	3	6 (7.9)
Total	24	52	76	24	52	76

*Notes:* Primary/lower secondary education and unemployed/secretary/lower technical jobs carer (low SES); secondary education (including Mature for Austria) and pensioner/middle manager occupation/business owner; tertiary education (high SES). SES for cared-for person: primary education and income below €1,700 (Austria) or self-reported “low income” (Slovenia; low SES); secondary education (except Matura for Austria) and monthly income between €1,700 and €2,500 (Austria) or self-reported “medium income” (Slovenia; middle SES); upper secondary education or monthly income above €2,500 (Austria) or self-reported “high income” (high SES).

### Data Collection

Interviews were carried out between February and September 2019. Most interviews took place at the interviewees’ own home, and as a rule, each member of the dyad was interviewed separately, unless interviewees requested that the other dyad member be present during the interview.

The semistructured interviews used a topic guide with prompts and open-ended questions covering four areas: biographies before care, the moment of needing care, providing care, and paying/rewarding for care. The interviewer followed up on the answers provided with further questioning in a conversational manner. Interviews lasted between 20 and 70 min, and all were audio-recorded and transcribed verbatim in the original language. Sociodemographic information of each interviewee was gathered in a short questionnaire before each interview, and further contextual details of the interviewee were recorded as field notes during and immediately after each interview.

To account for the dyadic structure of the study, we used identical topic guides for both members of the dyad to capture parallel reflections on transitions to care and past life events or trajectories; employed an “embedded triangulation” (Solomon et al., 2018) by having the two members of each dyad report on one transition into caregiving. Topic guides were jointly developed in English by the whole research team and then back-translated and piloted in the language of the participants in each site.

### Data Analysis

Framework Analysis (Ritchie & Lewis, 2003) was applied for data analysis using MAXQDA software (Supplementary Document I). This analytical approach allows for the combination of deductive reasoning, to first establish a conceptually clear structure of themes, with inductive theory-building analysis, by including codes and expanding themes that stem from the transcribed interviews (Fereday & Muir-Cochrane, 2006). The initial coding frame was derived from the theoretical framework described above and then filled in and modified by codes arising from the interviews (e.g., family conflict, intergenerational exchanges; Bradley et al., 2007). This mix of deductive concept-based and inductive data-driven coding facilitated homogeneity in the analysis of both national data sets (Gibbs, 2018) and avoided bias arising from identifying only themes from the framework. For example, Pathway 3 (*continued and sustained exchanges of support*) arose from the narratives of dyads and was not originally present in the theoretical framework derived from the literature.

To ensure validity and following Eisikovits and Koren (2010) and Hudson et al. (2020), we first coded and analyzed each individual interview, before focusing on the dyad as a unit and systematically identifying consistencies, discrepancies, and omissions in their accounts. Considering the cross-language nature of the study (Squires, 2009), the coding was carried out in the original language by two coders in each national team to limit language barriers between researchers and participants and maximize the sociocultural competence of each research team in relation to their study site. To ensure intercoder reliability within each country, the first batch of interviews was coded separately and codes compared. As for the between-country reliability, the theoretically developed original theme frame mentioned above was also meant to contribute to intersite validity. In addition, both national research teams maintained regular exchanges as new codes emerged and a code lexicon was developed to ensure intercoder consistency. Some, albeit few codes were exclusive of one national data set, reflecting the uniqueness of each context or findings. Thematic matrices summarizing the information for analysis (Supplementary Document I) were developed in English and jointly analyzed by both national teams. A comprehensive audit trail of decisions, including field notes and memos of meetings, was kept throughout.

## Results

The results are organized around the four distinct pathways that emerged from the analysis: specific turning point events in life, cumulative processes of caregiving, continued and sustained exchanges, and linked lives. For each pathway, we present also similarities and differences across the two countries, SES, and gender.

### Pathway 1: Turning Point Events in Life

This pathway refers to transitions in family or employment trajectories of dyads—termed turning points in their lives—that were associated with family caregiving.

Chiefly among the key events with a direct influence on care decisions were those connected with family trajectories. These included carers’ own divorces, which led them to find themselves alone when needs arose (e.g., without a spouse to take up care), or those of their parents, meaning that carers were raised by their grandparents for whom they would later care for. These also included the carers’ own children moving out and thus freeing carers from having multiple caring obligations. Among these family trajectories were interviewees who as the youngest (female) child had remained longer in the parents’ house and had begun already then to provide some form of support:

I am the youngest, was at home for the longest time and am involved in almost all of the decisions, have been almost everywhere jointly responsible, regardless of what was done. (A13, Austrian female caregiver, medium SES)

This was credited with creating a precedent and expectation for care later on, even when carers had already moved out of their parents’ house and did not reside in the vicinity at the onset of needs.

Another key transition pertained to cohabitation. Some carers never left the family home as their parents’ house was large enough to accommodate them and they had been unable or unwilling to find their own lodging. Others had returned to their parents’ home, chiefly motivated by financial reasons after moving away in search of work, or after divorces:

Yes … simply, wife and I separated. I used to work here [name omitted due to anonymity] and had only the basic wage … if you are alone … you are struggling. Until it was two of us, there was no problem. But when you are alone and you have to pay 300€ per month it is … oh God. (LP15, Slovene male caregiver, medium SES)

This cohabitation singled these adult children as “natural carers” when needs arose.

There were also references to transitions related to the labor market. A number of interviewees had just retired or were on prolonged sick leave when needs arose and this enabled the take-up of the caring role.

Some of the above-mentioned transitions were mediated by gender, but less so by SES. For instance, the timing of leaving the parents’ home was intertwined with the interviewees’ role as the daughter who helped around in the house, while the brothers had left as soon as possible to work somewhere else. Similarly, those who had never left their parents’ home were mostly women. For male caregivers, transitions associated with withdrawal from the labor market featured more prominently as pathways into caregiving. Cohabitation or residing very close by was more often found in rural areas among multigenerational farming families, which were present mostly in the Slovenian sample.

### Pathway 2: Caregiving as a Cumulative Process

A second pathway into caregiving reflected cumulative processes triggered by life events that came to influence the transition to caregiving.

A number of interviewees cited a personal history of weak labor market attachment (e.g., linked to several spells of sick leave) or having left the labor market altogether some time before (e.g., being inactive after long spells of unemployment) as the main factor leading to caregiving. When asked to reflect on the circumstances that led them to take up care, one interviewee offered this reflection:

And I have to say, that mine was classic woman’s employment in Austria, two part-time jobs, not at all corresponding to my education … but my mother isn’t to blame for this at all. This just resulted from my biography, that I have been with the children for a very long time and therefore re-entered into the workforce late. (A25, Austrian female caregiver, high SES)

For another female caregiver in Austria, taking up caregiving as her main occupation paid and being paid by the care allowance was framed against her own personal history of weak labor market attachment intertwined with frequent episodes of poor health.

The divorces mentioned earlier did not merely triggered a transition in the family trajectories of carers that led to caregiving—Pathway 1. They were accompanied by or caused a deterioration of the financial situation of would-be carers. This, in turn, was a strong driving force behind cohabitation, as hinted by the words of the Slovenian carer LP15 above.

While the cumulative processes narrated by interviewees mostly referred to growing disadvantages that led to caregiving, there were exceptions to this. This cumulative pathway also included two daughters who had previously worked as professional carers. In both cases, interviewees framed their current condition of family caregivers as a continuation of this previous (professional) caring experience. The previous roles seem to have elicited renewed expectations within the family as to the interviewees’ overall suitability as carers, including their perceived greater technical skill or ability to navigate the care system:

I have been … at the time we needed help, I have been employed in a nursing home as a nurse. And when mother had problems, when she needed care and everything, there was no one, that could come at a moment notice, and there was a waiting line [for care services]. So, I was called on, to provide care three times a day, seven days a week. (LP56, Slovene female caregiver, high SES)

Cohabitation following family dissolution, albeit referred by both male and female caregivers, weighted more heavily in the pathways into caregiving of carers with low SES.

### Pathway 3: Continued and Sustained Exchanges of Support

This pathway inductively arose from the accounts of interviewees and included past and continuous exchanges of different forms of support within the dyad. These exchanges generated a moral “debt” or expectation to reciprocate with care at the present point in their life trajectories.

Quite a number of caregivers and cared-for persons referred to reciprocity as a clear and strong motivation for care, offering examples or linking current caregiving to concrete instances of support provided or received in the past. These past and very often continuous exchanges were of a varied nature including financial support and grandparenting. In some instances, these exchanges were linked to particular transitions or stages in life such as teenage pregnancy, divorces, or upbringing:

They’ve helped me a lot, with the divorce first of all. That I could go out from time to time or professionally. When attending school, he [her young child] was looked after until 16:30, afternoon childcare, which was great anyway. And often I didn’t get home before 17:30. And mum covered the hour. And that is what she is getting back now, I would say. (A50, Austrian female caregiver, high SES)

Yes, I raised him [grandchild] and that’s why he says “Yes, granny, you have done so much for me in my life, I will pay you back now. I’m here for you now.” (B28, Austrian female care receiver, average SES)

Most dyads were interviewed separately but there was a very high degree of agreement between both members on this reciprocal motivation. In two cases, this cumulative process was framed in relation to the generation to which the cared-for person belonged:

This is my solidarity contribution. He suffered during war times, partly his generation re-built this country, I was already born into a generation where I say “Ok, we had a nicer childhood and this is actually the thing that one can give back.” (A10, Austrian female caregiver, low SES)

This cumulative process resulting from reciprocal exchanges was more often found among dyads that enjoyed close relationships. There were exceptions to this rule though, as exemplified by one interviewee who downplayed her emotional attachment while still acknowledging the reciprocal exchange underlying her caregiving:

I would say a normal relationship. Nothing particular. Linked enough, I think, so parents helped in the beginning, also financially, me and my brother. And I guess we are returning this now in this way. (LP07, Slovene female caregiver, high SES)

These exceptions not only confirm the relevance of these reciprocal exchanges for caregiving in later stages in life, but they were separated from norms of filial support. In fact, several interviewees clearly stated that caregiving might not have been forthcoming in the absence of these past continued exchanges and support.

This pathway into informal caregiving was transversal to gender and country. Views on reciprocity were not stronger among individuals of lower SES. Indeed, this pathway was more frequent among higher socioeconomic groups.

### Pathway 4: Linked Lives and Life Trajectories of Others

In this pathway, we analyze transitions into caregiving that are affected by the choices, behaviors, and resources of other family members. In other words, how transitions into caregiving are affected by life trajectories of others in the family.

Family conflicts played an outsized role in the division of care within families. As a rule, these were not disputes between siblings, but rather between the cared-for person and other children than the carer. For example, one carer cited a previous disagreement that took place between her mother and a daughter-in-law, which caused the older brother to refrain from providing care. Although references to these conflicts were not frequent, when present they were referred to as particularly strong impediments for other relatives to provide care.

The “social convoys,” understood here as the web of relatives that could be called upon at the onset of need, also depended very much on the stage each sibling was in their employment, family, or health trajectories. Several siblings were not available as they still had children of their own to care for, or could provide only limited care in addition to the main carer because they resided far away, were still employed or had health problems: “I have work, my sister is ill and cannot help. She is three years younger, but she has multiple sclerosis” (LP23, Slovene female caregiver, high SES).

The life trajectories of other siblings were referred to as pathways to caregiving across gender lines. However, these seemed to have a greater significance as a pathway into caregiving for carers of lower SES. This could signal that financial resources could more easily make up for gaps in the availability of other family members in social convoys, that is, care services could be bought and replace unavailable relatives.

## Discussion

The starting point for this study was to use tenets of life course theories to build workable categories for research on pathways *into* family caregiving. Based on a theoretical framework that encompasses key life events, cumulative processes, and linked lives, we found four nonmutually exclusive pathways into caregiving (Table 2). The first pathway pertained to transitions occurring in family or work trajectories of carers that expanded the possibilities for caregiving later in life. These included transitions into single living by carers, following divorce, or cohabitation with parents. The work transitions that influenced caregiving were associated with more conventional work trajectories along the life stages, namely, transitions to retirement. The second pathway referred to cumulative processes triggered by key life events usually during adulthood that later influenced transitions into caregiving. For instance, interrupted careers due to childcare led to a weak labor market attachment later in life that in turn facilitated the transition into a caregiving role, or previous roles as professional carers that may endow on these individuals care specific abilities or expectations for care later in life. This accumulation was distinguished from the cumulative processes described in Pathway 3, which were defined around continuous reciprocal exchanges of support that were underpinned by what Yeandle called “the negotiated and historical development of relationships between families” (Yeandle, 1996, p. 524). This included the web of interdependencies and reciprocal exchanges forged in lived family trajectories as well as in caring relationships (Rodrigues, 2020). There were other ways in which pathways into family caregiving were shaped by the ebb and flow of family relationships, particularly the life trajectories of other family members. This was evident in Pathway 4, defined around the linked lives of siblings and social convoys (Kahn & Antonucci, 1980). In this pathway, we find examples of how the life trajectories of siblings affected who was available to provide care, but also how strained family relationships or conflicts affected the implicit decision of who becomes a carer within families.

**Table 2.** Summary and Framework of Pathways Into Family Caregiving

Pathways	Life course tenet	Description	Overlapping pathways	Examples
1. Turning point events in life	Life events and transitions	Transitions in family or work trajectories of carers that place particular individuals in a position to take up care later on	These transitions are the starting point of cumulative processes: accumulating advantages/disadvantages (Pathway 2) and triggering downward support from cared-for persons earlier in the life course (Pathway 3)	Divorces followed by cohabitation with parents; retirement of carers
2. Caregiving as a cumulative process	Cumulative processes	Cumulative advantages and (mostly) disadvantages shaped by institutional contexts	Associated with or triggered by earlier transitions in different domains (Pathway 1)	Deteriorating financial situation after divorces or weak labor market attachment; serial caring roles
3. Continued and sustained exchanges of support	Cumulative processes	Continued support (in-kind and financial) from cared-for persons earlier in the life course that generates an expectation to reciprocate with care later in the life course	Exchanges of support are linked to particular life course stages or transitions (e.g., after marriage/divorce) (Pathway 1), as well as cumulative processes of advantage/disadvantage (Pathway 2)	Upbringing; grandparenting; financial support in earlier life stages (e.g., around marriage/divorce of carers)
4. Linked lives and life trajectories of others	Linked lives	Life trajectories of other family members (who are not members of the dyad) influence the composition of “social convoys”	The trajectories of other relatives affect the family networks that enabled exchanges of support (Pathway 3)	Family conflicts; family or employment obligations of other relatives (potential carers)

As mentioned above, these pathways overlapped. Some key life events or transitions (e.g., “traumatic” events in the life of carers) triggered cumulative processes of disadvantage as well as opportunities for cohabitation that offered greater scope for downward intergenerational support, which reinforced family ties and generated a moral debt. Similarly, family conflicts often reconfigured the webs of support within families that generated ties that bind. The overlapping nature of these pathways is coherent with the interdependency of life trajectories across different domains stressed by life course theory (cf. Alwin, 2012).

Pathway 2 refers to more conventional cumulative processes such as those defined by CAD theories (Crystal & Shea, 1990; Dannefer, 2003) and mediated by SES and gender. An example of the former is cohabitation as a coping strategy following family dissolution for lower-income individuals. Conversely, Pathway 3 describes a cumulative process specific to family care and close to Finch and Mason’s (1993) concept of intrafamily support as a manifestation of responsibilities that develop over time and are part of a negotiation process within families underlined by power and control. They highlight that “the conditions under which people live their lives make it more likely that parents and children will develop commitments to each other” (Finch & Mason, 1993, p. 168). The cumulative processes reflected the historical times and institutions in which the biographies of dyads and the cumulation processes unfolded (Elder et al., 2003; O’Rand, 1996), namely, one characterized still by the male breadwinner model in both Austria and Slovenia and deeply gendered employment trajectories. A life course analysis applied to trajectories into caregiving thus also highlights a structuralist view of inequalities that underpin caregiving. There were ample examples of how agency was constrained by normative and institutional aspects of social order, either within the family, through the moral obligation to reciprocate in two deeply familialistic societies, or through biographies that were shaped by gendered norms and roles.

The study has some limitations that are worth considering. The sample analyzed, and indeed the analytical framework proposed, focuses on intergenerational care to older people underpinned by kinship relations. It does not include spousal care nor other types of caregiving (e.g., to children with disability or working-age parents) or those linked to specific conditions (e.g., dementia) that may have different pathways into care (cf. Hamilton & Adamson, 2013). Recall bias is a possibility when interviewees look back at transitions into caregiving. To minimize this possibility, the narratives within dyads were checked for consistency when interviewed separately and these were found to concur as to the recollection of events. Unlike spousal care, transition into intergenerational family care is often abrupt rather than gradual (Seltzer & Li, 1996), which also contributes to limit recall bias. Dyads were mostly interviewed separately, precluding us from observing reactions to contradictions and contrast their recollections. At the same time, this approach enabled each dyad member to narrate the story in her/his own terms and likely avoided social desirability bias (e.g., when describing family conflicts or exchanges) and “quantitative dominance” in which one member of the dyad might be seen as the “natural” interlocutor for a given subject (Hudson et al., 2020). This approach also allowed us to establish with little reasonable doubt the reciprocal nature of the cumulative family exchanges narrated by dyads.

Life trajectories are also very much molded by the place and era in which they take place. We applied our framework to a diverse sample of individuals in two otherwise relatively similar countries insofar as caring arrangements and types of familialism (Saraceno, 2016). A more in-depth exploration of country or institutional differences was beyond the scope of this study. Future research could explore how pathways into caregiving may diverge from those described here, for example, in settings defied by more differentiated forms of familialism, individualistic mores, or life trajectories in dual breadwinner societies. For now, this study has contributed to expanding a body of research on life course and caregiving that has been mostly focused on Anglo-Saxon countries. Similarly, care cultures and institutions are not static and pathways into caregiving may well vary across different cohorts, reflecting, for example, greater economic independence of women or different family formation patterns. This would be a worthwhile future research avenue to explore, as would possible intersections with other measures of social location, namely, race and ethnicity (reflecting population characteristics in the countries analyzed, our sample was homogeneous in these latter aspects).

Applying life course lenses to care has until now focused on the sequencing of care episodes or/and the role that caregiving plays in the cumulation of disadvantages during and in the postcare period (Fast et al., 2020; King & Pickard, 2013; Seltzer & Li, 1996). Our main contribution to this field lies in a framework for the analysis of pathways at the other bookend of care, that is, into caregiving. This has the potential to show patterns of persistence and cumulative disadvantage in some carers, linked to serial caring roles, previous weak labor market attachment, and limited financial resources. This is important from a policy standpoint, situating caregiving with previously existing disadvantages (e.g., in income and health) and pointing to the need for policies to acknowledge these disadvantages. There is indeed some evidence that family caregiving, particularly of intense form, may be becoming increasingly concentrated on disfavored groups (Rodrigues & Ilinca, 2021). Provision of health insurance and pension credits to carers may limit part of the consequences of these disadvantages at the time of caring. In comparison to Europe, federal legislation supporting family carers in the United States (the Family and Medical Leave Act of 1993) still lags considerably behind (Heymann et al., 2008) and as health care is dependent on the employer, caregiving may play a disproportionate role in widening inequalities. To fully address these cumulative processes, however, carers would benefit from policy interventions aimed at sustaining income and health throughout the life course aimed at the general population.

With this study, we aimed to demonstrate the opportunity and indeed the relevance of applying the tenets of life course theory to fully understand pathways into intergenerational caregiving within families. In doing so, we contribute to furthering the research in this field and to the arguments made by others in favor of considering care as a distinct life course domain, while at the same time suggesting that life course lenses may render visible the circumstances leading to caregiving.

## Supplementary Material

gbac024_suppl_Supplementary_Material

## References

[CIT0001] Alwin, D. F . (2012). Integrating varieties of life course concepts. The Journals of Gerontology, Series B: Psychological Sciences and Social Sciences, 67(2), 206–220. doi:10.1093/geronb/gbr146 22399576 PMC3307990

[CIT0002] Barczyk, D., & Kredler, M. (2019). Long-term care across Europe and the U.S.: The role of formal and informal care. Fiscal Studies, 40(2), 329–373. doi:10.1111/1475-5890.12200

[CIT0003] Bradley, E. H., Curry, L. A., & Devers, K. J. (2007). Qualitative data analysis for health services research: Developing taxonomy, themes, and theory. Health Services Research, 42(4), 1758–1772. doi:10.1111/j.1475-6773.2006.00684.x 17286625 PMC1955280

[CIT0006] Carmichael, F., & Ercolani, M. G. (2016). Unpaid caregiving and paid work over life-courses: Different pathways, diverging outcomes. Social Science & Medicine (1982), 156, 1–11. doi:10.1016/j.socscimed.2016.03.020 27010581

[CIT0007] Crystal, S., & Shea, D. (1990). Cumulative advantage, cumulative disadvantage, and inequality among elderly people. The Gerontologist, 30(4), 437–443. doi:10.1093/geront/30.4.437 2394380

[CIT0008] Crystal, S., Shea, D. G., & Reyes, A. M. (2017). Cumulative advantage, cumulative disadvantage, and evolving patterns of late-life inequality. The Gerontologist, 57(5), 910–920. doi:10.1093/geront/gnw056 27030008 PMC5881660

[CIT0009] Dannefer, D . (2003). Cumulative advantage/disadvantage and the life course: Cross-fertilizing age and social science theory. The Journals of Gerontology, Series B: Psychological Sciences and Social Sciences, 58(6), 327–337. doi:10.1093/geronb/58.6.s327 14614120

[CIT0010] Dannefer, D . (2020). Systemic and reflexive: Foundations of cumulative dis/advantage and life-course processes. The Journals of Gerontology, Series B: Psychological Sciences and Social Sciences, 75(6), 1249–1263. doi:10.1093/geronb/gby118 30295844

[CIT0011] Elder, G. H. Jr . (1985). Perspectives on the life course. In G. H. Elder (Ed.), Life course dynamics: Trajectories and transitions, 1968–1980 (pp. 23–49). Cornell University Press.

[CIT0012] Elder, G. H. Jr., Johnson, M. K., & Crosnoe , R. (2003). The emergence and development of life course theory. In J. T. Mortimer & M. J. Shanahan (Eds.), Handbook of the life course (pp. 3–19). Kluwer Academic/Plenum.

[CIT0013] Eisikovits, Z., & Koren, C. (2010). Approaches to and outcomes of dyadic interview analysis. Qualitative Health Research, 20(12), 1642–1655. doi:10.1177/1049732310376520 20663940

[CIT0014] Eurobarometer . (2007). Health and long-term care in the European Union. In Special Eurobarometer 283 (pp. 1–247). European Commission. https://data.europa.eu/data/datasets/s657_67_3_ebs283?locale=en

[CIT0015] Eurostat . (2015). People in the EU. Who are we and how do we live. Eurostat statistical books. Publications Office of the European Union. https://ec.europa.eu/eurostat/documents/3217494/7089681/KS-04-15-567-EN-N.pdf/8b2459fe-0e4e-4bb7-bca7-7522999c3bfd

[CIT0016] Fast, J., Keating, N., Eales, J., Kim, C., & Lee, Y. (2020). Trajectories of family care over the lifecourse: Evidence from Canada. Ageing & Society, 41(5), 1145–1162. doi:10.1017/S0144686X19001806

[CIT0017] Fereday, J., & Muir-Cochrane, E. (2006). Demonstrating rigor using thematic analysis: A hybrid approach of inductive and deductive coding and theme development. International Journal of Qualitative Methods, 5(1), 1–11. doi:10.1177/160940690600500107

[CIT0018] Finch, J., & Mason, J. (1993). Negotiating family responsibilities. Routledge.

[CIT0019] Genda, Y., Kondo, A., & Ohta, S. (2010). Long-term effects of a recession at labor market entry in Japan and the United States. The Journal of Human Resources, 45(1), 157–196. doi:10.3368/jhr.45.1.157

[CIT0020] Gibbs, G. R . (2018). Analyzing qualitative data (Vol. 6). Sage.

[CIT0046] Graham, H. (2002). Building an inter-disciplinary science of health inequalities: the example of life course research. Social Science & Medicine, 55(11), 2005–2016. doi:10.1016/s0277-9536(01)00343-4 12406467

[CIT0021] Hamilton, M. G., & Adamson, E. (2013). Bounded agency in young carers’ lifecourse-stage domains and transitions. Journal of Youth Studies, 16(1), 101–117. doi:10.1080/13676261.2012.710743

[CIT0022] Heymann, J., Earle, A. & Hayes, J. (2008). The work, family, and equity index: How does the United States measure up? Institute for Health and Social Policy at McGill University.

[CIT0023] Hudson, N., Law, C., Culley, L., Mitchell, H., Denny, E., Raine-Fenning, N. (2020). Conducting dyadic, relational research about endometriosis: A reflexive account of methods, ethics and data analysis. Health, 24(1), 79–93. doi:10.1177/1363459318786539 29978723 PMC6873217

[CIT0024] Kahn, R. L., & Antonucci, T. C. (1980). Convoys over the life course: Attachment, roles, and social support. In P. B. Baltes & O. Brim (Eds.), Life-span development and behavior (Vol. 3, pp. 254–283). Academic Press.

[CIT0025] Keating, N., Eales, J., Funk, L., Fast, J., & Min, J. (2019). Life course trajectories of family care. International Journal of Care and Caring, 3(2), 147–63. doi:10.1332/239788219X15473079319309

[CIT0026] Kramer, B. J., & Lambert, J. D. (1999). Caregiving as a life course transition among older husbands: A prospective study. The Gerontologist, 39(6), 658–667. doi:10.1093/geront/39.6.658 10650675

[CIT0027] King, D., & Pickard, L. (2013). When is a carer’s employment at risk? Longitudinal analysis of unpaid care and employment in midlife in England. Health & Social Care in the Community, 21(3), 303–314. doi:10.1111/hsc.12018 23356685

[CIT0028] Landman, T . (2008). Issues and methods in comparative politics. An introduction. Routledge.

[CIT0029] Larkin, M., Henwood, M., & Milne, A. (2019). Carer‐related research and knowledge: Findings from a scoping review. Health and Social Care in the Community, 27, 55–67. doi:10.1111/hsc.12586 29846020

[CIT0030] Merton, R. K . (1968). The Matthew effect in science. The reward and communication systems of science are considered. Science (New York, N.Y.), 159(3810), 56–63. doi:10.1126/science.159.3810.56 5634379

[CIT0031] Michaud, P. C., Heitmueller, A., & Nazarov, Z. (2010). A dynamic analysis of informal care and employment in England. Labour Economics, 17(3), 455–465. doi:10.1016/j.labeco.2010.01.001

[CIT0032] Moen, P., & DePasquale, N. (2017). Family care work: A policy-relevant research agenda. International Journal of Care and Caring, 1(1), 45–62. doi:10.1332/239788217X14866284542346 28825046 PMC5557024

[CIT0033] Moen, P., & Sweet, S. (2004). From ‘work–family’ to ‘flexible careers’. Community, Work & Family, 7(2), 209–226. doi:10.1080/1366880042000245489

[CIT0034] O’Rand, A. M . (1996). The precious and the precocious: Understanding cumulative disadvantage and cumulative advantage over the life course. The Gerontologist, 36(2), 230–238. doi:10.1093/geront/36.2.230 8920094

[CIT0035] Ritchie, J., & Lewis, J. (2003). Qualitative research practice: A guide for social science students and researchers. Sage Publications.

[CIT0036] Rodrigues, R . (2020). Caring relationships and their role in users’ choices: A study of users of Direct Payments in England. Ageing & Society, 40(7), 1469–1489. doi:10.1017/S0144686X19000035

[CIT0037] Rodrigues, R., & Ilinca, S. (2021). How does she do it all? Effects of education on reconciliation of employment and informal caregiving among Austrian women. Social Policy & Administration, 55(7), 1162–1180. doi:10.1111/spol.12706

[CIT0038] Roth, D. L., Perkins, M., Wadley, V. G., Temple, E. M., & Haley, W. E. (2009). Family caregiving and emotional strain: Associations with quality of life in a large national sample of middle-aged and older adults. Quality of Life Research, 18(6), 679–688. doi:10.1007/s11136-009-9482-2 19421895 PMC2855243

[CIT0039] Saraceno, C . (2016). Varieties of familialism: Comparing four southern European and East Asian welfare regimes. Journal of European Social Policy, 26(4), 314–326. doi:10.1177/0958928716657275

[CIT0040] Schmid, T., Brandt, M., & Haberkern, K. (2012). Gendered support to older parents: Do welfare states matter? European Journal of Ageing, 9, 39–50. doi:10.1177/0958928716657275 28804406 PMC5547314

[CIT0041] Seltzer, M. M., & Li, L. W. (1996). The transitions of caregiving: Subjective and objective definitions. The Gerontologist, 36(5), 614–626. doi:10.1093/geront/36.5.614 8942105

[CIT0042] Solomon, D. N., Hansen, L., & Baggs, J. G. (2018). It’s all About the relationship: Cognitively intact mother–daughter care dyads in hospice at home. The Gerontologist, 58(4), 625–634. doi:10.1093/geront/gnw263 28329822

[CIT0043] Squires, A . (2009). Methodological challenges in cross-language qualitative research: A research review. International Journal of Nursing Studies, 46(2), 277–287. doi:10.1016/j.ijnurstu.2008.08.006 18789799 PMC2784094

[CIT0044] Van Houtven, C. H., Coe, N. B., & Skira, M. M. (2013). The effect of informal care on work and wages. Journal of Health Economics, 32(1), 240–252. doi:10.1016/j.jhealeco.2012.10 23220459

[CIT0045] Yeandle, S . (1996). Work and care in the life course: Understanding the context for family arrangements. Journal of Social Policy, 25(4), 507–527. doi:10.1017/S0047279400023928

