# Supplementary material for: Life Course Pathways Into Intergenerational Caregiving
Source: J Gerontol B Psychol Sci Soc Sci. 2022 Feb 5;77(7):1305–14. doi: 10.1093/geronb/gbac024 (PMC9255941; doi:10.1093/geronb/gbac024)
Supplement: gbac024_suppl_Supplementary_Material [file gbac024_suppl_supplementary_material.pdf]

Supplemental Document I: Detailed account of analysis using Framework Analysis

The analysis using Framework Analysis involved four stages. In the first stage each national research team gained in-depth knowledge of their own data through coding of the transcripts using MAXQDA software. The theoretical propositions deducted from the framework presented above guided the initial coding frame for all transcripts. The themes included *transitions, cumulative processes* and *joint lives*. Each theme was continuously reviewed and populated by codes arising from the interviews (e.g. family conflict, intergenerational exchanges) (Bradley et al., 2007). This mix of deductive concept-based and inductive data-driven coding aimed to ensure all elements referring to the topic were captured. In a second stage, data was summarized using thematic matrices, with each cell synthetizing information on a code (column) and individual (row). In a third stage, codes were examined for consistency and variation across the sample (e.g. patterns or outliers), aggregating codes where relevant to reflect higher abstraction and analysing the remaining events and processes mentioned by each dyad to identify main pathways and possible variation by SES and gender patterns in each country. In the fourth and final stage of the analysis both teams drew conclusions from the patterns and exceptions found in the data.
